# Confocal imaging of biomarkers at a single-cell resolution: quantifying 'living' in 3D-printable engineered living material based on Pluronic F-127 and yeast *Saccharomyces cerevisiae*

**DOI:** 10.1186/s40824-022-00337-8

**Published:** 2022-12-21

**Authors:** Bojan Žunar, Taiga Ito, Christine Mosrin, Yoshiyuki Sugahara, Hélène Bénédetti, Régis Guégan, Béatrice Vallée

**Affiliations:** 1 Centre de Biophysique Moléculaire (CBM), CNRS, UPR 4301, University of Orléans and INSERM, 45071 Orléans, Cedex 2 France; 2 Department of Chemistry and Biochemistry, Laboratory for Biochemistry, Faculty of Food Technology and Biotechnology, University of Zagreb, 10000, Zagreb, Croatia; 3 Department of Applied Chemistry, Faculty of Science and Engineering, Waseda University, Tokyo, 169-8555 Japan; 4 Global Center for Advanced Science and Engineering, Faculty of Science and Engineering, Waseda University, Tokyo, 169-8555 Japan; 5 Institut des Sciences de la Terre d’Orléans (ISTO), UMR 7327, CNRS-Université d’Orléans, 1A Rue de la Férollerie, 45071 Orléans, Cedex 2 France

**Keywords:** Engineered living materials, 3D-bioprinting, Bioink, Hydrogel, Pluronic F-127, *Saccharomyces cerevisiae*

## Abstract

**Background:**

Engineered living materials (ELMs) combine living cells with non-living scaffolds to obtain life-like characteristics, such as biosensing, growth, and self-repair. Some ELMs can be 3D-printed and are called bioinks, and their scaffolds are mostly hydrogel-based. One such scaffold is polymer Pluronic F127, a liquid at 4 °C but a biocompatible hydrogel at room temperature. In such thermally-reversible hydrogel, the microorganism-hydrogel interactions remain uncharacterized, making truly durable 3D-bioprinted ELMs elusive.

**Methods:**

We demonstrate the methodology to assess cell-scaffold interactions by characterizing intact alive yeast cells in cross-linked F127-based hydrogels, using genetically encoded ratiometric biosensors to measure intracellular ATP and cytosolic pH at a single-cell level through confocal imaging.

**Results:**

When embedded in hydrogel, cells were ATP-rich, in exponential or stationary phase, and assembled into microcolonies, which sometimes merged into larger superstructures. The hydrogels supported (micro)aerobic conditions and induced a nutrient gradient that limited microcolony size. External compounds could diffuse at least 2.7 mm into the hydrogels, although for optimal yeast growth bioprinted structures should be thinner than 0.6 mm. Moreover, the hydrogels could carry whole-cell copper biosensors, shielding them from contaminations and providing them with nutrients.

**Conclusions:**

F127-based hydrogels are promising scaffolds for 3D-bioprinted ELMs, supporting a heterogeneous cell population primarily shaped by nutrient availability.

**Graphical Abstract:**

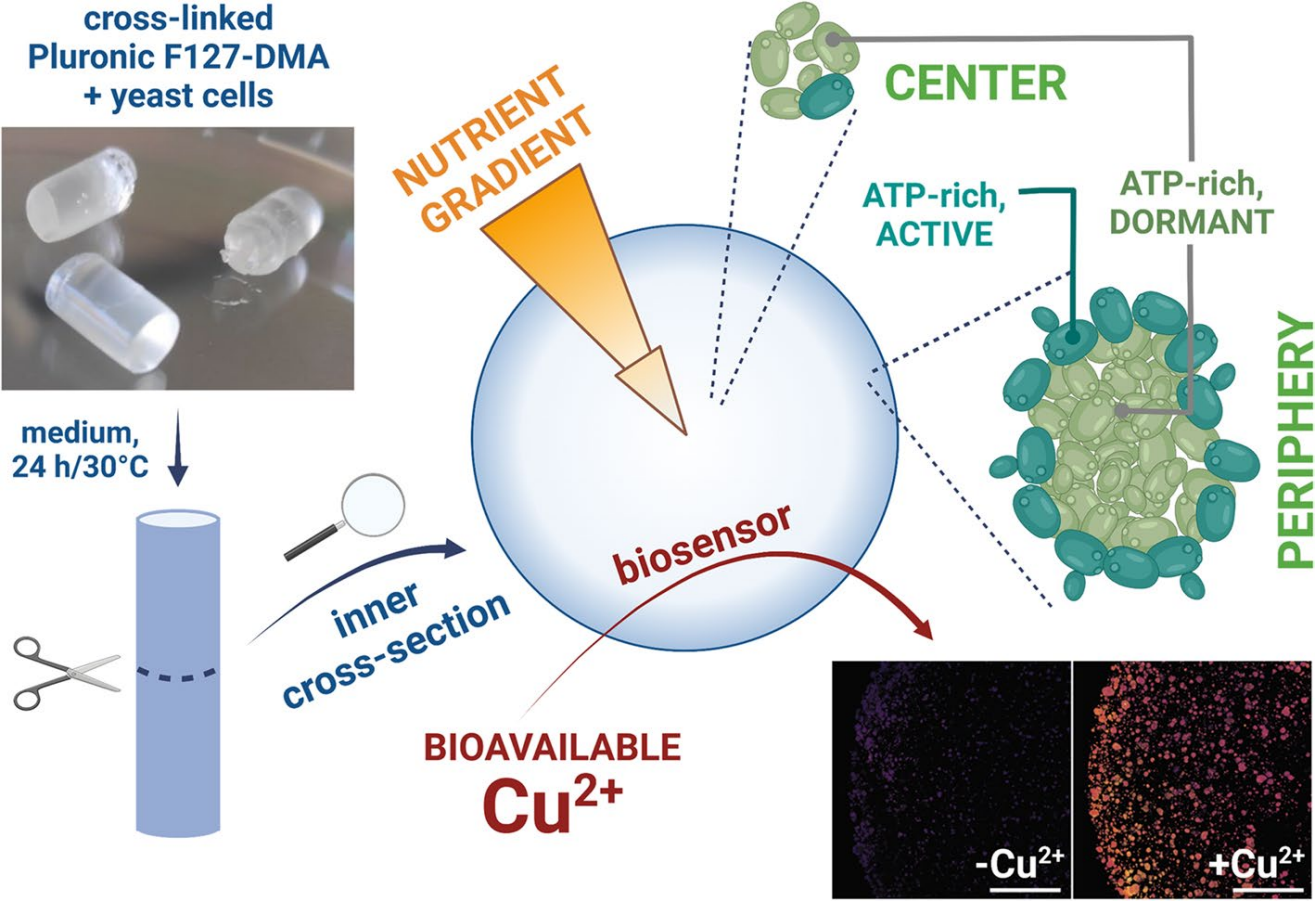

**Supplementary Information:**

The online version contains supplementary material available at 10.1186/s40824-022-00337-8.

## Background

Inspired by natural materials, such as wood and bone, engineered living materials (ELMs) combine living cells with a non-living scaffold to obtain life-like traits, e.g. biosynthesis, sensing, growth, and self-repair [1]. While initially developed for tissue and organ engineering, ELMs today employ bacteria, yeast, and algae [2] to produce innovative bioproducts, e.g. self-oxygenating wound patches [3], light-based bioelectricity [4], living-tattoo biosensors [5], self-healing concrete [6], next-generation biofertilizers [7], and artificial corals [8].

The only promising scaffolds for ELM are those that promote cell survival. Among suitable materials, hydrogels are particularly noteworthy, allowing inward and outward diffusion of nutrients and metabolites [9]. Moreover, by being malleable, some hydrogels serve as bioinks in additive manufacturing, i.e. 3D-bioprinting [10]. These hydrogels are mostly polymers synthesized by living organisms, such as agar, agarose, alginin, and κ-carrageenan, but suffer drawbacks, e.g. too high melting temperatures or cation-dependent cross-linking [1].

One alternative to 3D-printable natural hydrogels is Pluronic F127 (Poloxamer 407), a long linear molecule with a central polypropylene glycol (PPG) block flanked by two polyethylene glycol (PEG) blocks [11]. As such, central part of the F127 molecule is more hydrophobic than its ends. This property allows Pluronic F127 to exhibit reverse gelling. Indeed, an aqueous solution of Pluronic F127 is liquid at 4 °C. However, as the temperature rises, F127 molecules self-organize into micelles with a hydrophobic core, and at above 20 °C at 25% w/w solution, the micelles self-order into a crystal cubic structure [12, 13], thus producing liquid crystal [14]. Interestingly, such material can be used as a shear-thinning, 3D-printable hydrogel [15] that can be extruded at room temperature, supporting high cell viability. Moreover, its rheological and gelling properties can be further modulated with inorganic nanosheets [16]. To fix the hydrogel into a permanent non-dissolvable gel state, ends of the F127 molecules are often functionalized with small molecules, e.g. dimethacrylate (DMA) or bisurethane methacrylate (BUM), which can be chemically cross-linked using photoinitiator and UV-light [17, 18]. F127-DMA is used when polymer integrity and cell retention are paramount, e.g. in bioprocesses. On the other hand, F127-BUM can be degraded by proteases and has lower cell retention time, which makes it interesting for biomedical applications [19].

Yeast *S. cerevisiae* is a model eukaryote with the GRAS status (generally regarded as safe), widely used in traditional biotechnology in beer-, wine- and bread-making [20]. Moreover, some of its strains show a well-established probiotic effect [21]. Being well-studied and easy to genetically modify [22–24], *S. cerevisiae* is also readily chosen as a workhorse in modern bioprocesses aimed at the biosynthesis of biofuels, recombinant proteins, and biologically active small molecules [25], such as caffeine, resveratrol, morphine, and artemisinin [26]. Finally, it serves as a chassis for many eukaryotic whole-cell biosensors [27]. Thus, 3D-bioprinted *S. cerevisiae*-ELMs promise to revolutionize existing biotechnological niches and open novel ones [28].

Previous studies documented metabolically active microcolonies of *S. cerevisiae* cells in 3D-printed F127-DMA and F127-BUM hydrogels, thus demonstrating functional biotechnologically-relevant ELMs [29, 30]. However, they did not characterize energetic state of the embedded cells, i.e. their ATP levels, cell survival once the cells stop dividing, nor the permeability of these materials to the outside molecules. On the other hand, these studies noted a gradient in microcolony size, undersized cells [30], and near-anaerobic conditions in hydrogels [19]. Moreover, they pointed out the challenges of recovering and characterizing cells from chemically cross-linked hydrogels, which they overcame by dehydrating, using supercritical CO_2_, and flash-freezing the hydrogels.

Alternatively, hydrogel-embedded cells can be equipped with fluorescent proteins that quantify intracellular metabolites in single cells, i.e. with genetically-encoded, single fluorescent protein-based biosensors [31]. These proteins are engineered to allosterically respond to specific small molecules, whose binding modulates protein’s fluorescence. Some such proteins are simply mutated GFPs, e.g. pH-responsive pHlourin [32], while others are GFP variants fused with an analyte-binding polypeptide, i.e. sensing domain, that tugs on the amino acid side chains near the chromophore when the domain binds the analyte [33]. Such conformational change affects the chromophore's excitation coefficient, protonation state or quantum yield [34], thus producing either intensiometric or ratiometric biosensors. Yeast *S. cerevisiae* is especially convenient for expressing such biosensors as it is possible to integrate the required genes at precise chromosomal loci, thus standardizing their expression and copy number. The constructed yeast strains can then serve to study cell-hydrogel interactions directly in situ, at a single-cell level.

In an intensiometric biosensor, analyte binding increases or decreases protein's fluorescence but does not perturb protein's excitation spectra [35]. Thus, a strong signal marks either an unusually high concentration of uninduced reporter or an average concentration of induced reporter. On the other hand, in ratiometric biosensors, analyte binding disproportionally changes sections of the excitation spectra, which favors quantitative imaging, as each analyte concentration produces a unique excitation spectrum. With such biosensors, the analyte concentration is measured by exciting the sample with two wavelengths and calculating the ratio at their emission maxima. Only with such genetically-encoded ratiometric fluorescent biosensors, the metabolism can be explored in real-time and non-destructively, within the original and undisturbed spatial context.

Only a subset of biologically relevant analytes can be detected with allosterically-regulated ratiometric fluorescent biosensors [31]. However, even non-allosterically-regulated ratiometric biosensors could be used analogously if they were based on a differential expression of two fluorescent proteins. One example would be a classical whole-cell biosensor that reacts to an analyte of interest by inducing transcription of the reporter gene while constitutively expressing an additional reporter to normalize the induced signal. We have recently developed one such biosensor to quantify bioavailable copper [36], a ubiquitous and essential heavy metal that is toxic at high concentrations and whose environmental presence thus needs to be strictly monitored [37]. Of course, the successful application of this construct in the hydrogel-embedded cells would demonstrate that Pluronic F127-based ELMs can be used as easily deployable environmental biosensors.

In this work, we present a widely applicable methodology to study alive, metabolically active yeast cells in hydrogels. Moreover, this methodology allowed us to account for the relative position of the cells within the scaffold. We embedded biosensor-expressing cells in thick F127-DMA hydrogel plugs (5.4 mm in diameter) and imaged them with confocal microscopy, measuring their cell density, ATP levels, cytosolic pH, and diffusion of copper ions throughout the plug, at a single-cell level. We determined thickness of the hydrogel layer that supported rich cell growth and uncovered differences in energy levels and growth phase between and within intact microcolonies, which pointed out the profound effect of the nutrient gradient. Finally, we demonstrated that F127-DMA hydrogel is a promising ELM for easy deployment of whole-cell biosensors, creating, as a proof of concept, a copper-sensing living material.

## Methods

### Media and growth conditions

*E. coli* was grown overnight at 37 °C, either in 2xYT liquid media (16.0 g tryptone, 10.0 g yeast extract, 5.0 g NaCl per 1 l of media) or on LB solid media (10.0 g tryptone, 5.0 g yeast extract, 5.0 g NaCl, 15.0 g agar per 1 l of media), supplemented with 100 mg/l ampicillin.

*S. cerevisiae* was grown at 30 °C/180 rpm in a chemically defined medium (6.70 g/l Difco Yeast nitrogen base without amino acids, 20.0 g/l glucose, 0.77 g/l MP Bio drop-out mixture, and 20.0 g/l agar for solid media). In the imaging experiments, Difco's YNB was replaced with Formedium's LoFlo YNB without amino acids, folic acid, and riboflavin (Formedium, Hunstanton, United Kingdom). In the experiments with copper, Difco's YNB was replaced with Formedium's YNB without amino acids and copper. Copper was supplemented as CuSO_4_ (Merck, Germany). Strains expressing ymNeongreen [38], QUEEN-2 m [39], and sfpHlourin [40] were grown in -his medium, while strain expressing copper biosensor [36] was grown in -ura medium.

### Strain construction

Plasmids constitutively expressing ymNeongreen and sfpHlourin were constructed in *E. coli* NEB Stable with NEB HiFi Assembly Kit (New England Biolabs, Evry, Frace) and verified by Sanger sequencing. Oligonucleotide synthesis and Sanger sequencing were outsourced to Eurofins Genomics (Konstanz, Germany). Details of the constructions are given in Supplementary material 1.

Together with MTP 3067 [39], constructed plasmids were linearized with PstI and integrated as multiple copies into the *his3Δ1* locus of BY 4742 [41]. Correct targeting and multiple integration were verified by colony PCR with NEB OneTaq MasterMix. The BY 4742 strain carrying chromosome-integrated pCu2 was obtained from Žunar et al. [36].

### F127-DMA synthesis

F127-DMA was synthesized as in Saha et al. [29]. Shortly, 10.5 g of Pluronic F127 (Sigma-Aldrich, Germany) was vacuum-dried at 50 °C/24 h, mixed with 82.5 ml of anhydrous toluene (Fujifilm Wako Pure Chemical Corporation, Tokyo, Japan) under an N_2_ atmosphere and then mixed with 2.04 ml of triethylamine. The mixture was cooled down to 0 °C and combined with a mixed solution of 7.5 ml of anhydrous toluene and 1.44 ml of methacryloyl chloride (90%; TCI, Fukaya City, Japan). Addition to this mixture was performed dropwise, under stirring, through a funnel, over 30 min, and was followed by stirring the mixture at 0 °C/1 h, and then at room temperature for 24 h or more under an N_2_ atmosphere. Then, a vacuum-filtration was performed at 40 °C. The collected filtrate was concentrated under reduced pressure. Next, 90 ml of toluene was added, and the mixture was warmed to 40 °C. Filtration and decompression steps were repeated three times, and the filtrate was concentrated under reduced pressure (50 hPa) with the addition of 30 ml of toluene. With an excess of 210 ml of diethyl ether, a precipitate was formed, which was separated by centrifugation (4400 rpm/15 min, repeated twice). The white precipitate, i.e. F127-DMA, was dried under ambient conditions for 12 h and then under reduced pressure at 40 °C for 24 h. Dried F127-DMA was stored in amber-glass containers at 4 °C.

Rheological experiments were performed on a modular compact rheometer MCR 102 (Anton Paar, Austria), with a parallel plate (25 mm in diameter).

### Hydrogel preparation

**Fig. 1:**
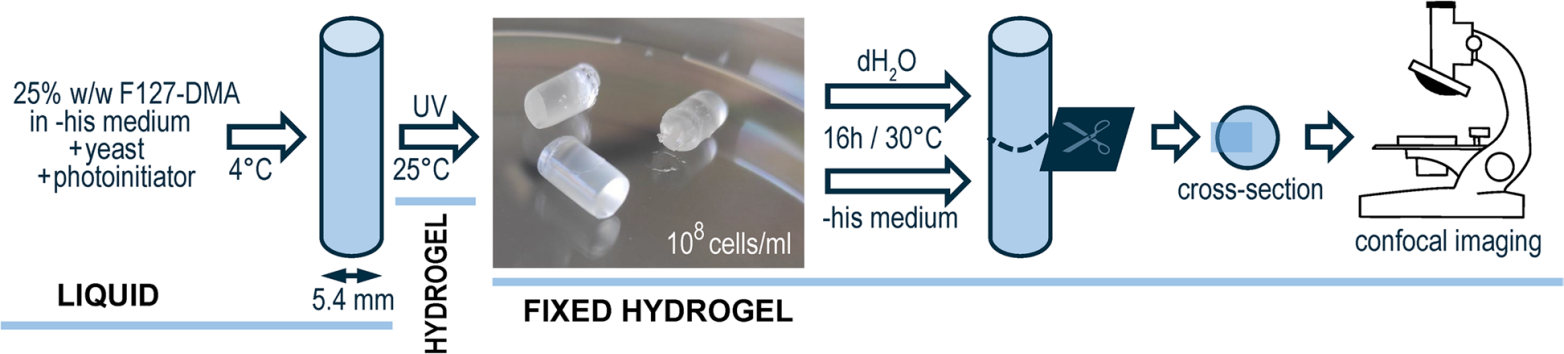
Preparation of the hydrogel plugs. The 25% w/w F127-DMA was mixed with yeast cells and photoinitiator at 4 °C. The mixture was cast into the mold, incubated at 0 °C/30 min, incubated at 25 °C/30 min, cross-linked with 365 nm/120 s, and incubated at 25 °C/30 min. The hydrogel plug was taken from the mold and incubated at 30 °C/16 h in deionized water or chemically defined yeast medium, after which it was cut in half, and its inner cross-section imaged

F127-DMA was dissolved to a 25% w/w solution in a chemically defined medium by using a rotating wheel overnight at 4 °C. Yeasts were grown at 30 °C/180 rpm to 10^7^ cells/ml, washed twice with sterile deionized water and mixed on ice with 25% w/w F127-DMA and 2-hydroxymethyl propiophenone (0.1%), which served as a photoinitiator (Fig. 1). The mixture was used to fill standard PCR tubes, which served as molds, and incubated at 0 °C/30 min and then at 25 °C/30 min. To cross-link the hydrogels, the tubes were irradiated with 365 nm/120 s using a 300-lm UV torch (Windfire, Shenzhen, China) from a distance of 30 cm, after which the tubes were incubated at 25 °C/30 min and cut in half by scalpel. The lower part of the PCR tube was discarded, and the cross-linked hydrogel plug was pushed out of the upper half. Finally, the plug was incubated in sterile water or chemically defined media at 30 °C/16 h, cut in half to expose the inner cross-section and imaged by confocal microscopy.

### Confocal imaging

Immediately after plug bisection, one-half of the plug was mounted in an 8-well Lab-Tek chambered #1.0 borosilicate coverglass system (Nunc, New York, USA), submerged in liquid, and immobilized. Imaging was performed on a ZEISS LSM 980 confocal microscope with Airyscan 2 (Carl Zeiss, Oberkochen, Germany). All experiments were performed in triplicate. Figures show the most representative micrographs for each condition. The lines in graphs below micrographs present a smoothed conditional mean obtained by local polynomial regression fitting (loess smoothing), with a span of 0.1, as implemented in the R “stats” package.

Cross-sections were imaged under a dry 2.5 × objective and microcolonies under a dry 20 × objective. The ymNeongreen plugs were excited with a 488 nm laser (3% strength at 2.5 ×, 1% strength at 20 ×), and emissions were gathered in the 499–553 nm range. The QUEEN-2m and sfpHlourin plugs were excited with 488 nm and 405 nm lasers (10% strength at 2.5 ×, 5% strength at 20 × for QUEEN-2 m; 2% and 3% strength at 2.5 ×, 2% strength at 20 × for sfpHlourin), and emissions were gathered in the 499–553 nm range. The pCu2 plugs were excited with 488 nm laser (5% strength at 2.5 ×, 5% strength at 20 ×) and 561 nm lasers (5% strength at 2.5 ×, 2% strength at 20 ×), and emissions were gathered in the 500–550 nm and 588–637 nm ranges, respectively.

Microcolony micrographs were Airyscan-processed with Zeiss Zen Blue software. Additionally, the extended depth of focus was calculated for the ymNeongreen-expressing microcolonies. When required, signal ratios were calculated with Fiji [42], as described in Takaine [43].

Control cells in 2-D-deoxyglucose and D-glucose solutions (Merck, Germany) were incubated at 30 °C/30 min in 2% aqueous solutions of appropriate sugars, resuspended in 25% w/w F127 dissolved in 2% solution of appropriate sugars, and imaged. Control cells with a cytosolic pH of 5.5 and 7.0 were prepared by permeabilization with 10 mg/ml digitonin (Across Organics, Geel, Belgium) as in Orij et al. [44], incubated at 30 °C/30 min in an appropriate citrate–phosphate buffer, resuspended in 25% w/w F127 dissolved in appropriate citrate–phosphate buffer, and imaged.

### Excitation spectra and ratiometric calibration

Cells were grown to 10^7^ cells/ml and disrupted with FastPrep FP120 Cell Disrupter (Qbiogene, Illkirch, France) as in Botman et al. [45]. For QUEEN-2m, cells were disrupted in 0.1 M KH_2_PO_4_/K_2_HPO_4_ buffer (pH 7.4) supplemented with 0.4 g/l MgCl_2_, and cell lysate was centrifuged at 21,000 g/4 °C/15 min. Next, 5 µl of supernatant was mixed with 35 µl of ATP solution in a black 384-well low-volume microplate with a flat bottom (Corning, New York, USA) to obtain 40 µl of mixture of the required ATP concentration. The experiment was performed in five parallels. For sfpHlourin, cells were disrupted in 0.01 M KH_2_PO_4_/K_2_HPO_4_ buffer (pH 7.4) supplemented with 0.4 g/l MgCl_2_, and cell lysate centrifuged at 21,000 g/4 °C/15 min. Next, 5 µl of supernatant was mixed with 35 µl of citrate–phosphate buffers of varying pH in a 384-well low-volume microplate with a flat bottom. Each pH value was measured in five parallels.

Clariostar Plus (BMG Labtech, Ortenberg, Germany) was preheated to 30 °C, and excitation spectra were measured by monitoring emission at 520 nm following excitation in the range 390–495 nm, with a step of 1 nm. Ratiometric calibration was performed first by exciting at 410/15 nm, using a 465 nm dichroic filter, and monitoring emission at 520/15 nm, and then by exciting at 480/15 nm, using a 500 nm dichroic filter, and monitoring emission at 520/15 nm. Data were exported to.xlsx format and processed in the R programming environment [46].

### Alphafold structural predictions

Alphafold-predicted structures were calculated with ColabFold [47, 48] under default settings, with three recycling iterations, using MMseqs2 to search UniRef and environmental sequences.

## Results

### Characterization of Pluronic F127-DMA

The Pluronic F127-DMA was synthetized as described in the Methods section. The ^1^H NMR of F127-DMA showed the average block length of polypropylene oxide (degree of polymerization, DP) of 59 and the average block length of polyethylene oxide (DP) of 104, with the total molecular weight of 11,524 g mol^−1^. The degree of DMA functionalization was about 55%, as calculated by comparing the measured integrated value to the theoretical one, by considering if all the PEO end groups were converted to methacrylate. Thus, the synthesized F127-DMA had 55% of the terminal PEO moieties functionalized by DMA.

We used rheological measurements to assess the temperature dependence of F127-DMA storage modulus (G') and loss modulus (G''). Through them, we determined that the sol–gel transition temperature of F127-DMA is 19 °C. We also distinguished between three F127-DMA states: sol (viscous) state (small G' and G''), softly entangled gel (rapidly increasing G' and G''), and hard gel state (plateauing G' and G'') (Supplementary material 2).

### Growth and morphology

We used confocal microscopy to investigate how cross-linked F127-based hydrogels affected yeast *S. cerevisiae*. For this purpose, we constructed a yeast strain constitutively expressing green fluorescent protein ymNeongreen, grew it in a chemically defined standard yeast medium, and embedded obtained cells in F127-DMA-based hydrogel. In this way, we obtained 5.4 × 10 mm plugs of cross-linked F127-DMA (25% w/w, dissolved in chemically defined medium) that per ml contained 10^8^ cells constitutively expressing ymNeongreen (Fig. 1). To investigate how cells adapted to the hydrogel environment, we then incubated those plugs at 30 °C/16 h in deionized water or chemically defined medium, cut the plugs in half to expose their inner cross-section and imaged them to observe the local cell density.

**Fig. 2:**
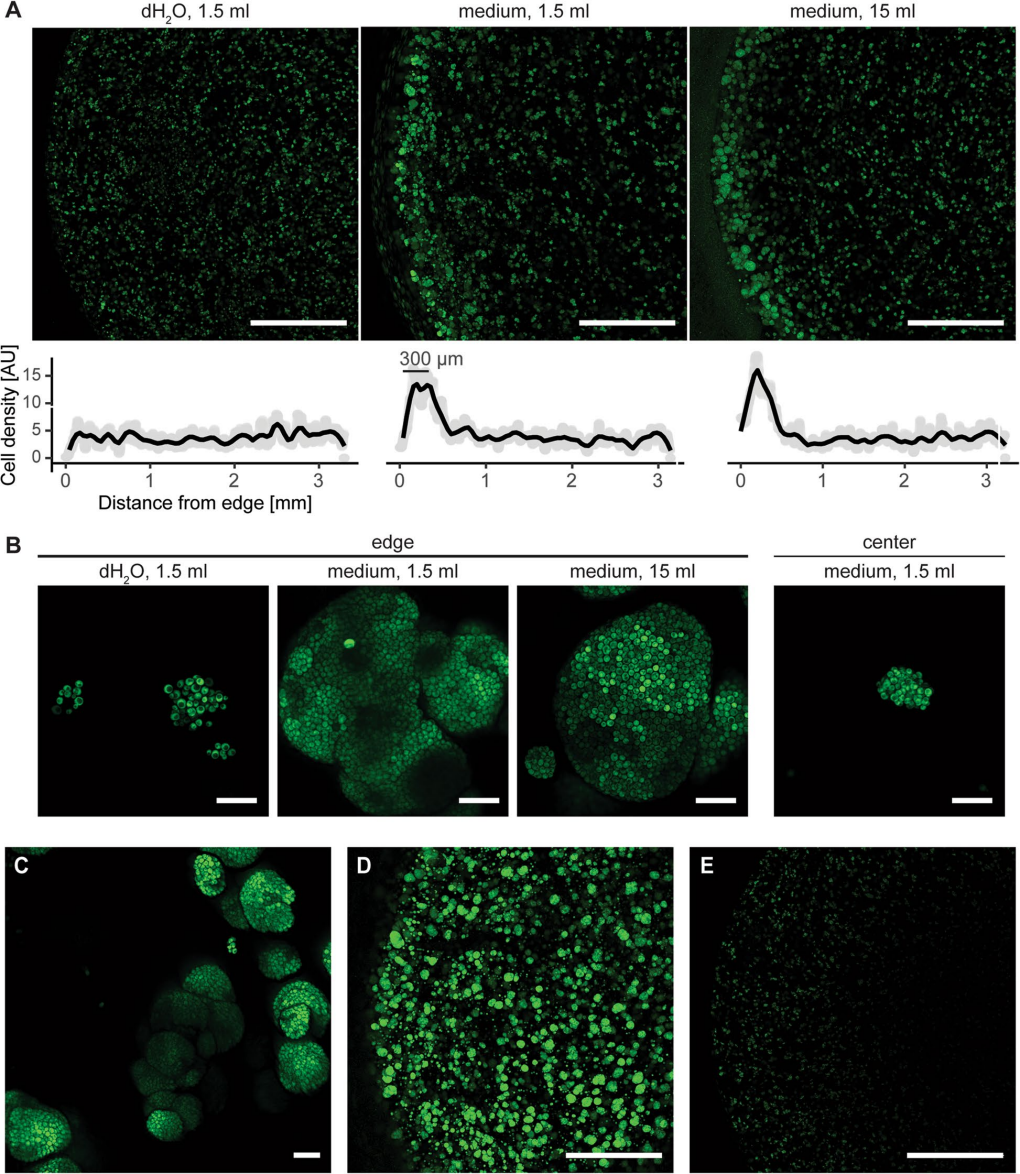
Hydrogel plugs with yeast cells expressing ymNeongreen. **A** Inner cross-sections of the hydrogel plugs after overnight incubation in 1.5 ml of deionized water, 1.5 ml of medium, or 15 ml of medium. Graphs under the micrographs denote mean cell density, with each grey point averaging one-pixel-wide column and a black line outlining the moving average. **B** Micrographs of the yeast microcolonies at the edge and center of the plugs. **C** Superstructures formed by merging several microcolonies at the edge of the media-incubated plugs. **D** Surface of the uncut media-incubated plug. **E** Inner cross-section of the air-exposed plug. Scale bars: 1 mm in A, D, and E, and 20 µm in B and C

We readily detected cells in all plugs. However, the distribution of cells in the water-incubated plugs differed from their distribution in the medium-incubated plugs (Fig. 2A). Throughout all plugs, cells expressed fluorescent ymNeongreen, which, as newly-synthesized fluorescent proteins need to react with molecular oxygen to become fluorescent [49], indicated aerobic conditions within the hydrogel. However, while the cells were uniformly distributed in the water-incubated plugs, they grew four-fold denser at the edges of the media-incubated plugs. Looking from the edge towards the center of the media-incubated plug, the cell density sharply declined around the depth of 300–400 µm, to levels similar to those of the water-incubated plugs. The centers of both plugs had the same cell density. Interestingly, the plugs incubated in 1.5 ml and 15 ml media had the same density profile, suggesting that 1.5 ml of media suffices to reach the maximal cell density values. Lower density at the very edges of these plugs reflected cells' leakage into the medium. Thus, cells survived and divided in cross-linked F127-DMA plugs, but as hydrogel probably hindered the diffusion of external nutrients, they grew heterogeneously, thriving at the plug's edge.

Higher magnification uncovered that, by dividing, cells formed microcolonies (Fig. 2B), both at the edge and the center of the plugs. The microcolonies contained several dozens to several thousand cells, with the large microcolonies arising only at the edge of the media-incubated plugs. In contrast, smaller microcolonies sprung throughout the entire cross-section of both plugs. When nearby, microcolonies often merged, forming superstructures (Fig. 2C). To confirm that the nutrient gradient caused heterogeneous growth, we imaged the top surface of the uncut media-incubated plug, fully exposed to the medium throughout overnight incubation and thus unaffected by the nutrient gradient. Here, the cells formed large and small microcolonies evenly across the entire surface area (Fig. 2D). Thus, the cells suspended in hydrogel formed microcolonies whose size depended on the amount of available nutrients.

During incubation in water and media, the plugs retained their overall shape but increased their volume by 50%. To see if the swelling of the plug affected cells, we also imaged unsubmerged plug, left in the air at 30 °C/16 h. In the inner cross-section of this plug, the cells formed microcolonies as in the water-incubated plug, but the fluorescent signal weakened towards the center (Fig. 2E), suggesting ymNeongreen failed to mature, probably as it lacked molecular oxygen. Thus, cells benefited from liquid-induced hydrogel swelling, which increased nutrient and oxygen diffusion within the plug.

### ATP content

**Fig. 3:**
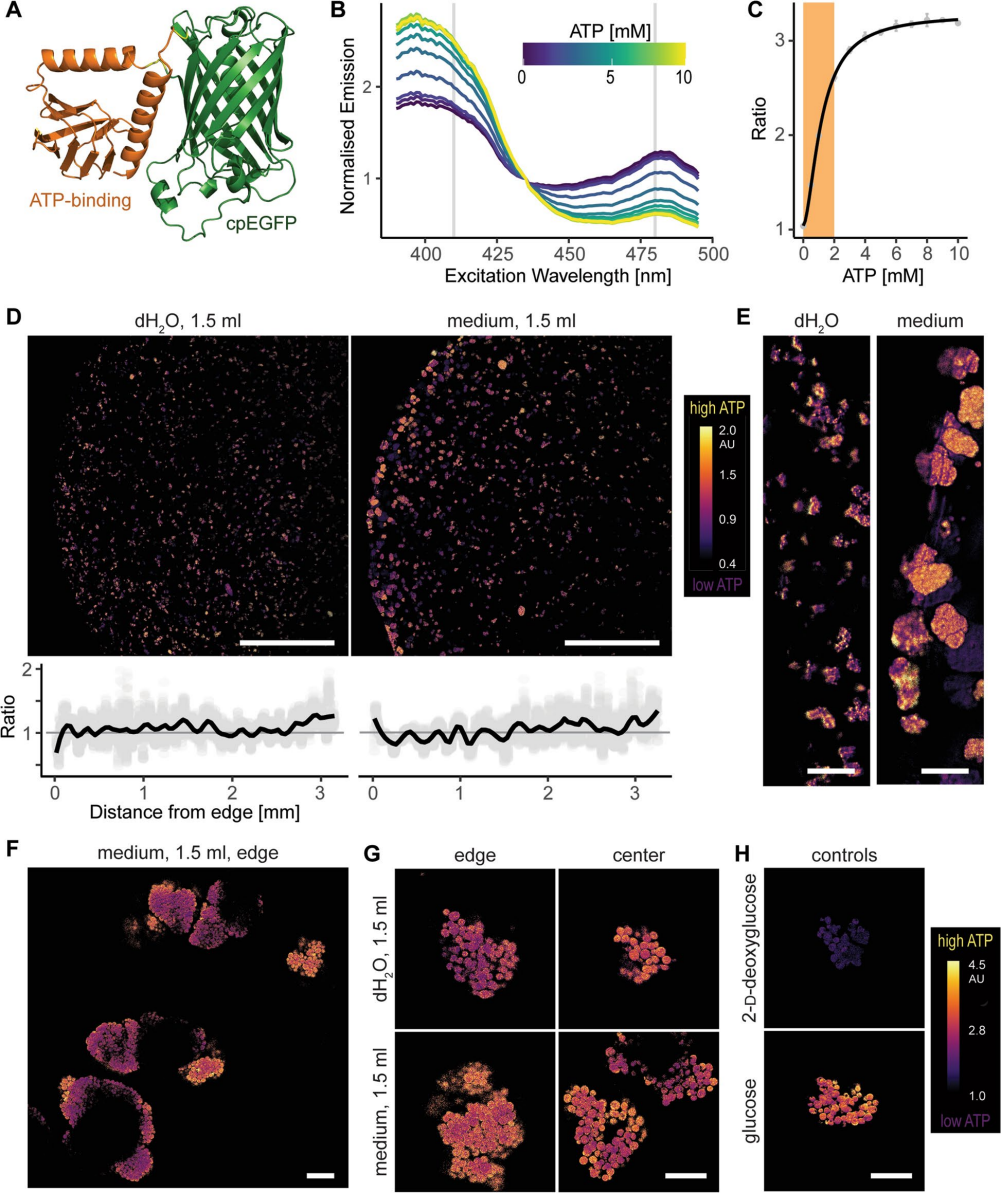
Single-cell ATP measurements in the hydrogel plugs carrying yeast cells that express QUEEN-2m, a genetically-encoded ratiometric GFP-based ATP biosensor. **A** Alphafold-predicted structure of QUEEN-2m, with annotated ATP-binding domain and circularly-permuted enhanced green fluorescent protein (cpEGFP). **B** Excitation spectrum of QUEEN-2m at different ATP concentrations. **C** Ratio of emissions at 520 nm following excitations at 410 and 480 nm, with orange shading marking the physiologically relevant ATP range and error bars denoting 95% confidence intervals. **D** Inner cross-sections of hydrogel plugs after overnight incubation in 1.5 ml of deionized water or media, with graphs showing the mean ratiometric signal. **E** Edge details of the micrographs in D. **F** Superstructure in the periphery of the media-incubated plugs. **G** Microcolonies at the edge and in the center of water- and media-incubated plugs. **H** Control ATP-depleted cells (incubated in 2-D-deoxyglucose [50]) and ATP-rich cells (incubated in D-glucose), with purple color denoting low ATP content and yellow color high ATP content. Scale bars: 1 mm in D, 100 µm in E, and 20 µm in F, G, and H

To further characterize physiology and metabolism of hydrogel-embedded microcolonies, we measured their ATP content. We assessed cell’s energy levels by constructing a strain constitutively expressing QUEEN-2m [39], a genetically encoded GFP-based ratiometric ATP biosensor made by fusing circularly permuted GFP with the ε-subunit of *Bacillus subtilis* (Fig. 3A). We confirmed that QUEEN-2m changed its excitation spectrum in the presence of ATP (Fig. 3B), and the ratiometric measurement of emissions at 520 nm upon excitations at 410 and 480 nm gave a near-linear response in the physiologically-relevant ATP range (0–2 mM, Fig. 3C), allowing us to estimate the ATP content of single cells for this range of concentrations.

Low magnification showed that the water- and media-incubated plugs (Fig. 3D) had similar, fairly non-changing ATP profiles that rise slightly towards the center of the plug. Predictably, microcolonies on the very edge of the media-incubated plug were larger and had more ATP than those at the edge of the water-incubated plug (Fig. 3E). Thus, despite differences in cell density, the ATP content of the plugs remained uniform throughout the cross-sections.

Higher magnification showed that ATP content varied within individual microcolonies and superstructures, with outer layers having more ATP than the central ones (Figs. 3F and G). Moreover, it confirmed that the microcolonies at the edge of the media-incubated plugs had more ATP than those at the periphery of the water-incubated plugs. Nevertheless, despite the differences, all cells registered as ATP-rich, compared to the ATP-depleted cells that served as an imaging control (Fig. 3H). Thus, despite minor differences in their ATP content, cells throughout microcolonies were ATP-rich, i.e. alive and well adjusted to their environment.

### Cytosolic pH

**Fig. 4:**
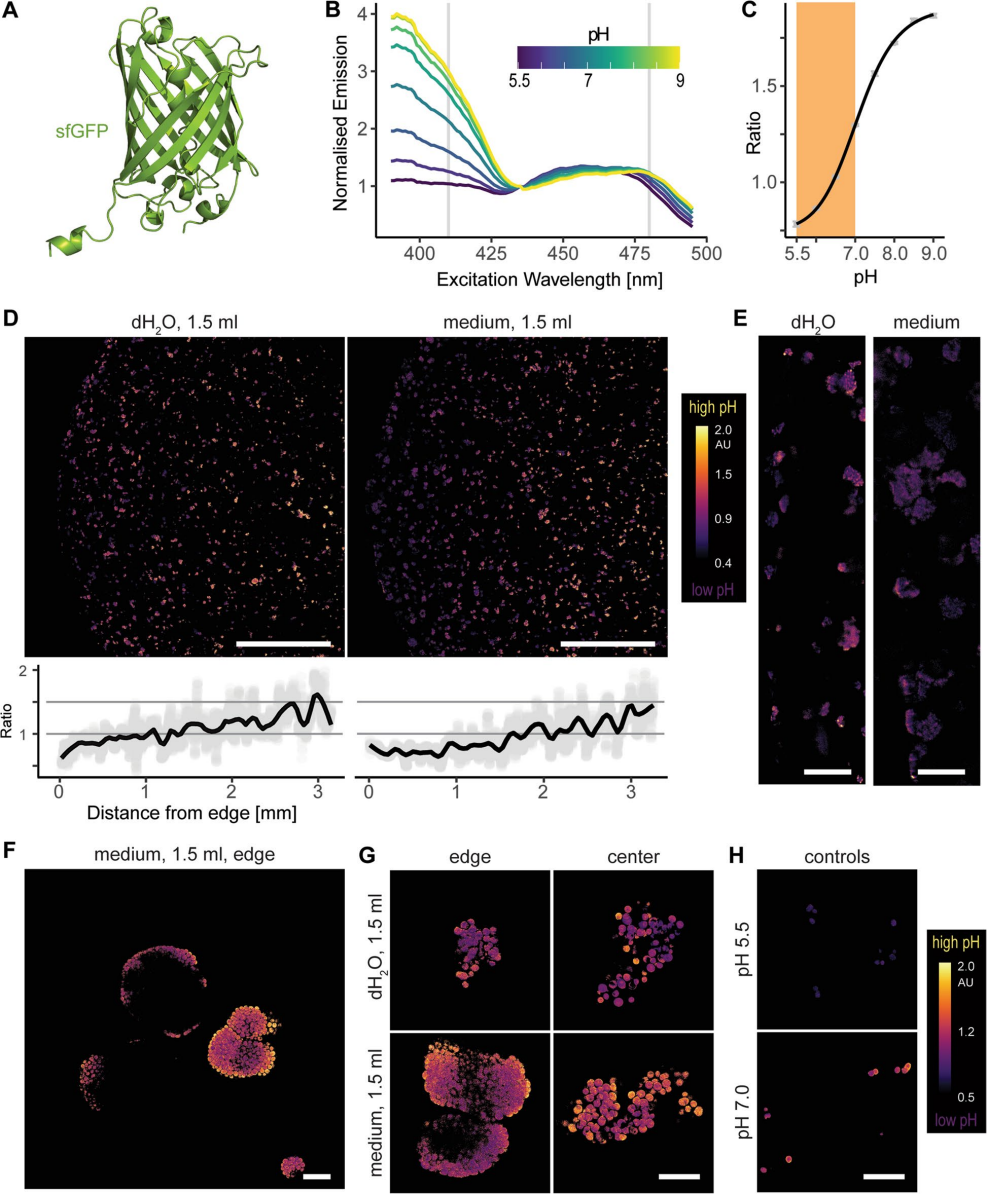
Single-cell measurements of cytosolic pH in the hydrogel plugs carrying yeast cells that express sfpHlourin, a genetically-encoded ratiometric GFP-based pH biosensor. **A** Alphafold-predicted structure of sfpHlourin, a superfolder green fluorescent protein (sfGFP) sensitive to pH changes. **B** Excitation spectrum of sfpHlourin at different pH. **C** Ratio of sfpHlourin emissions at 520 nm following excitations at 410 and 480 nm, with orange shading marking the physiologically relevant pH range and error bars denoting 95% confidence interval. **D** Inner cross-sections of the plugs incubated in 1.5 ml of water or media, with graphs denoting the mean ratiometric signal. **E** Edge details of the micrographs in D. **F** Superstructure in the periphery of the media-incubated plugs. **G** Microcolonies at the edge and in the center of water- and media-incubated plugs. **H** Permeabilized control cells incubated in pH 5.5 and pH 7.0, with purple color denoting lower cytosolic pH and yellow color higher cytosolic pH. Scale bars: 1 mm in D, 100 µm in E, and 20 µm in F, G, and H

Cells within microcolonies registered as ATP-rich, thus signaling they could be in either exponential (actively dividing) or stationary (latent) phase [51, 52]. To differentiate between these two alternatives, we measured cytosolic pH in single cells, which falls from pH 7 in the exponential phase to pH 5.5 in the stationary phase [53–55]. For this purpose, we used sfpHlourin (Fig. 4A; [40]), another genetically encoded GFP-based ratiometric biosensor. We confirmed sfpHlourin changed its excitation spectrum with pH (Fig. 4B), and the ratiometric measurements of emissions at 520 nm upon excitations at 410 nm and 480 nm gave a near-linear response for the physiologically relevant pH range (pH 5.5 – 7, Fig. 4C).

Low magnification seemingly uncovered a gradient of cytosolic pH (Figs. 4D and E), with the pH being lower at the periphery of the plugs and rising towards the center, although belatedly in the media-incubated plugs. However, higher magnification (Figs. 4F-H) showed that microcolonies throughout the media-incubated plugs had a brim of high-pH, nutrient-exposed cells that encircled a bulk of low-pH, nutrient-deprived cells. Thus, having access to nutrients, cells on the brim continued to actively grow, while the bulk of the cells entered stationary phase, having depleted the available nutrients. Thus, instead of reflecting a pH gradient along the cross-section, low magnification indicated a smaller percentage of the brim area in the large peripheral microcolonies. On the other hand, being smaller and slower-growing, microcolonies in the water-incubated plugs lacked the high-pH brim, as they were exposed to less nutrients. However, the plug's center still carried some high-pH, dividing cells, suggesting exponential growth, possibly delayed due to capped diffusion rates in the middle of the plug. Thus, cells grew throughout the plug, forming at the edge of the media-incubated plugs larger microcolonies with actively-dividing outer layer (high cytosolic pH, exponential growth phase) and a central bulk of dormant cells (low cytosolic pH, stationary phase).

### Diffusion

**Fig. 5:**
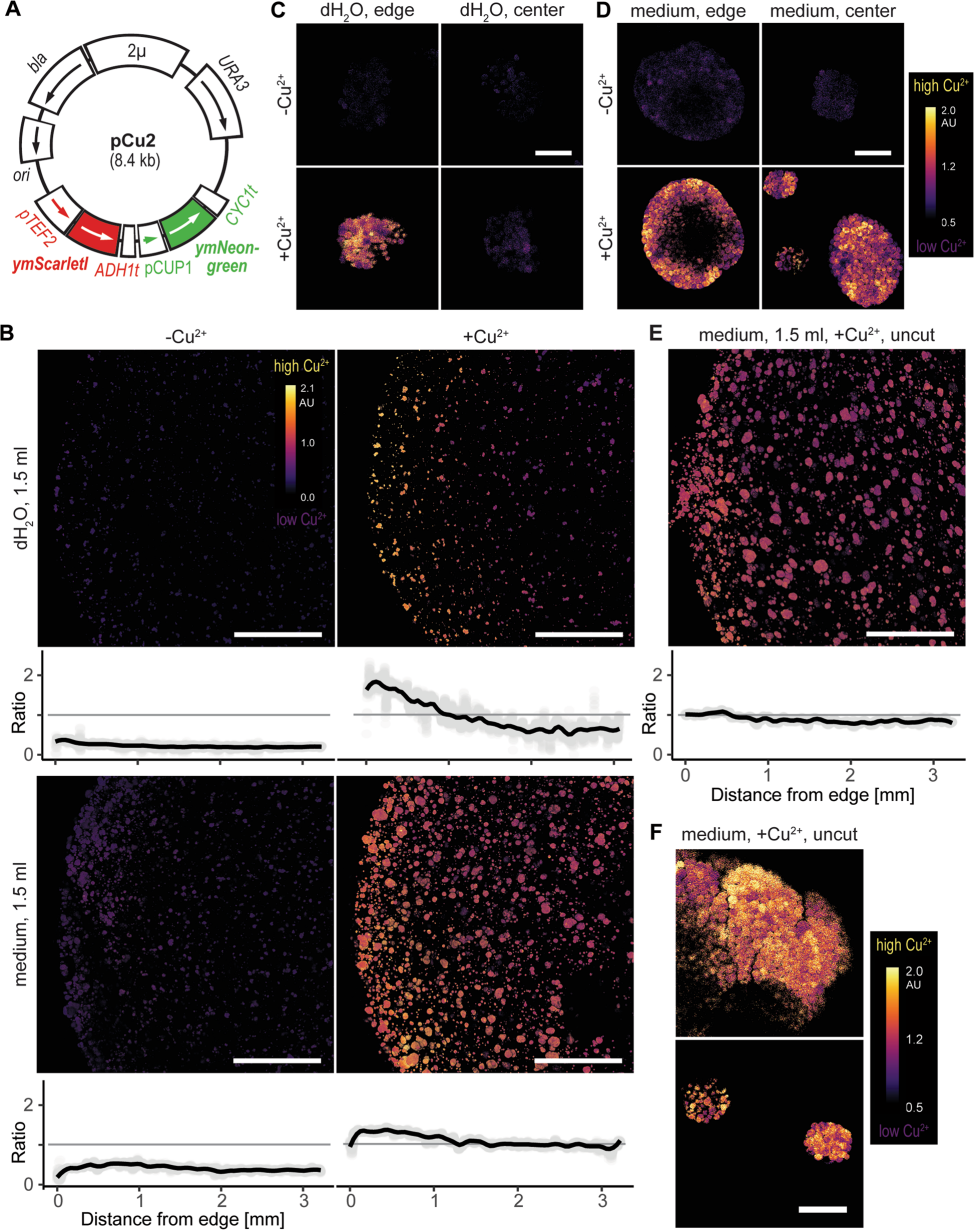
Copper detection in the hydrogel plugs carrying yeast cells expressing pCu2, a ratiometric copper biosensor. **A** Plasmid pCu2 encoding copper inducible green fluorescent protein ymNeongreen and constitutively expressed red fluorescent protein ymScarletI. Labelled regions encode β-lactamase (*bla*), pUC19 replication origin (*ori*), copper-inducible promoter (*pCUP1*), constitutive promoter (*pTEF2*), and yeast terminators (*ADH1t*, *CYC1t*). **B** Inner cross-sections of the plugs incubated in 1.5 ml of water or media, with or without 10 µM Cu^2+^, with graphs marking the mean ratiometric signal. **C** Microcolonies at the edge and center of the plugs incubated in water with or without 10 µM Cu^2+^. **D** Microcolonies at the edge and center of plugs incubated in media with or without 10 µM Cu^2+^. **E** Surface of uncut plug incubated in media with 10 µM Cu^2+^, with graph showing mean ratiometric signal. **F** Microcolonies at the surface of the uncut plug after overnight incubation in medium with 10 µM Cu^2+^, with purple color denoting low Cu^2+^ concentration and yellow color high Cu^2+^ concentration. Scale bars: 1 mm in B and E, and 20 µm in C, D, and F

To further characterize properties and biocompatibility of our ELM bioplugs, we tested whether small external molecules could reach the plug's center. For that purpose, we used yeast cells with chromosomally-integrated ratiometric copper biosensor pCu2, which encoded copper-inducible green fluorescent protein ymNeongreen and constitutively expressed red fluorescent protein ymScarletI (Fig. 5A). Thus, the diffusion of exogenous copper ions into the plug would raise the ymNeongreen/ymScarletI ratio within the cells. We embedded these cells in hydrogel plugs and incubated them at 30 °C/20 h in water or media, without Cu^2+^ or with 10 µM Cu^2+^, a concentration easily detected by our copper biosensor [36], yet biocompatible, i.e. non-toxic for yeast cells.

While copper-free plugs remained uninduced, plugs incubated with Cu^2+^ produced a strong signal. The signal weakened towards the plug's center (Fig. 5B), especially in water-incubated plugs, whose edges were the most induced. In the media-incubated plugs, the signal remained mostly homogenous, regardless of the distance from the edge. Interestingly, the signal was much stronger in water- than in media-incubated plugs, which high magnification micrographs confirmed further (Fig. 5C and D) and showed that without copper, all microcolonies produced faint ratiometric signals but emitted intense signals in its presence, except for those in the center of the water-incubated plugs. These results suggest that metabolic activity increased hydrogel's diffusibility. Finally, in larger microcolonies, outer layers emitted the strongest signal, probably because their cells were in exponential phase and continuing to responding to Cu^2+^. Thus, although hindered by hydrogel, Cu^2+^ could diffuse into the plugs, where it remained bioavailable [36] and readily detectable.

### ELM as a biosensor scaffold

The pCu2 results suggested that the plugs could also serve as scaffolds for deploying whole-cell biosensors. Ideally, such scaffolds would produce a uniform signal and not require post-processing, e.g. cutting. We tested the concept's feasibility by imaging the surface of the uncut plugs incubated in media with 10 µM Cu^2+^ (Figs. 5E and F). Indeed, the entire surface produced a uniform ratiometric signal, with both large and small microcolonies emitting intense fluorescence. Thus, F127-based hydrogels are promising scaffolds for ready-to-use portable whole-cell biosensors, supporting in natural environments in situ detection and direct read-out.

## Discussion

We studied the physiology of yeast cells embedded for 16 h in F127-DMA cross-linked hydrogel, by assessing four physiological markers: (i) cell growth and colony size, (ii) ATP content, as an indicator of the metabolic status, (iii) cytosolic pH, as an indicator of growth phase, and (iv) Cu^2+^ diffusion, as an indicator of hydrogel’s permeability. We showed that within this hydrogel, pervasive nutrient gradient shapes a heterogeneous ATP-rich population of exponential and stationary phase cells.

Our cross-linked F127-DMA hydrogel plugs carried fluorescent cells that expressed ymNeongreen or genetically encoded ratiometric biosensors for ATP, cytosolic pH, or copper. These fluorescent reporters allowed direct non-invasive high-resolution real-time imaging of live hydrogel-embedded cells. Moreover, being ratiometric, they nullified the effects of uneven reporter expression between cells. This approach could be expanded with additional genetically encoded ratiometric biosensors [33, 56], thus serving as a standard pipeline for assessing cell-scaffold interactions. Interestingly, a similar ratiometric strategy was recently applied to monitor yeast stress response in harsh industrial substrates, e.g. lignocellulosic hydrolysate [57]. Compared to the previously used F127-based 3D-bioprinted structures [19, 28–30], our hydrogel plugs were thicker, thus magnifying nutrient gradient and allowing us to quantify the gradients of colony size, ATP, and cytosolic pH. The plugs showed external metabolites can reach up to 2.7 mm inside the plug, and pointed to the plug thickness of 300 µm as the one supporting the maximal cell density (Fig. 2A), thus suggesting 600 µm as the maximal thickness for the 3D-bioprinted walls.

Given that fluorescent proteins become fluorescent only after reacting with oxygen [49], our confocal imaging pointed out (micro)aerobic conditions throughout the liquid-incubated plugs. On the contrary, the center of the unsubmerged plugs lacked strong fluorescence, suggesting a more hospitable environment for anaerobes, in line with Butelmann et al. [19]. Thus, oxygen diffuses more easily into liquid-incubated hydrogels, possibly due to the elution of non-cross-linked F127 molecules [18], which suggests that partial functionalization of Pluronic F127, in contrast to its complete functionalization, might benefit (micro)aerobes.

The measured ATP levels and cytosolic pH suggested that in larger microcolonies, cells differentiated, as they do on solid medium. Indeed, only the cells on the outer sphere of the microcolonies continued to divide, i.e. remained in the exponential phase. However, compared to colonies on solid medium, hydrogel-embedded microcolonies were spherical, suspended within the gel, and they even merged into higher-order superstructures. As such, they formed a novel type of multicellular yeast communities, adding to solid-medium colonies, biofilms, mats, stalks, flors, and flocks [58]. Interestingly, genetically modifying standard laboratory strains for different growth patterns within the hydrogel, e.g. for pseudohyphal growth [59], might affect ELM properties and give novel insights into yeast-scaffold interactions. Of course, throughout longer incubation and multiple rounds of growth, the microcolonies are expected to develop further, producing alive and dead zones [60] and changing their physiology in response to physical confinement [61]. These phenomena will be of interest in future studies.

By combining F127/medium-based hydrogels with pCu2 cells, we obtained a portable whole-cell copper biosensor that is self-sufficient, needing no external nutrients to produce the ratiometric signal. As such, this biosensor could replace time-consuming low-throughput methods for quantifying copper that are currently used by analytical laboratories [37], while also measuring only bioavailable copper, the only biologically relevant form of copper [36]. Thus, one could embed whole-cell biosensors into cross-linked hydrogel plugs, cut them into thin slices, incubate the slices directly in the sample (without the addition of nutrients), and measure fluorescence on one side of the slice for real-time in situ detection. However, for making a more general biosensor, the choice of the analyte might be crucial, as non-polar compounds partition into the middle of the F127 micelles [62–64]. On the other hand, such partitioning would shield yeast cells from harmful non-polar compounds present in polluted environments. Moreover, the hydrogel also shields yeast from contamination, as exogenous cells do not penetrate F127-based hydrogels [65]. Thus, F127/medium-based hydrogels are promising scaffolds for self-sufficient whole-cell biosensors.

## Conclusion

This study described the physiology of yeast cells in 3D-bioprintable F127-DMA-based cross-linked hydrogels, which serve as potential scaffolds in the next-generation ELMs. The cross-linked hydrogels were marked by a pronounced nutrient gradient but supported (micro)aerobic conditions and diffusion of molecules. Within them, yeast cells entered exponential and stationary phase and formed ATP-rich microcolonies of variable size, which was primarily dependent on nutrient availability. Finally, the hydrogels could nourish and shield whole-cell copper biosensors. Thus, F127-DMA-based hydrogels are promising scaffolds for 3D-bioprinted microbial living materials.

## Supplementary Information

**Additional file 1.**

**Additional file 2.**

## Data Availability

The datasets and materials used and analyzed during the current study are available from the corresponding author on reasonable request.

## Abbreviations

ELMs: Engineered living materials
DMA: Dimethacrylate
ATP: Adenosine triphosphate
GFP: Green fluorescent protein
