# Supplementary figures and images for: Confocal imaging of biomarkers at a single-cell resolution: quantifying 'living' in 3D-printable engineered living material based on Pluronic F-127 and yeast Saccharomyces cerevisiae
Source: Biomater Res. 2022 Dec 21;26:85. doi: 10.1186/s40824-022-00337-8 (PMC9769040; doi:10.1186/s40824-022-00337-8)

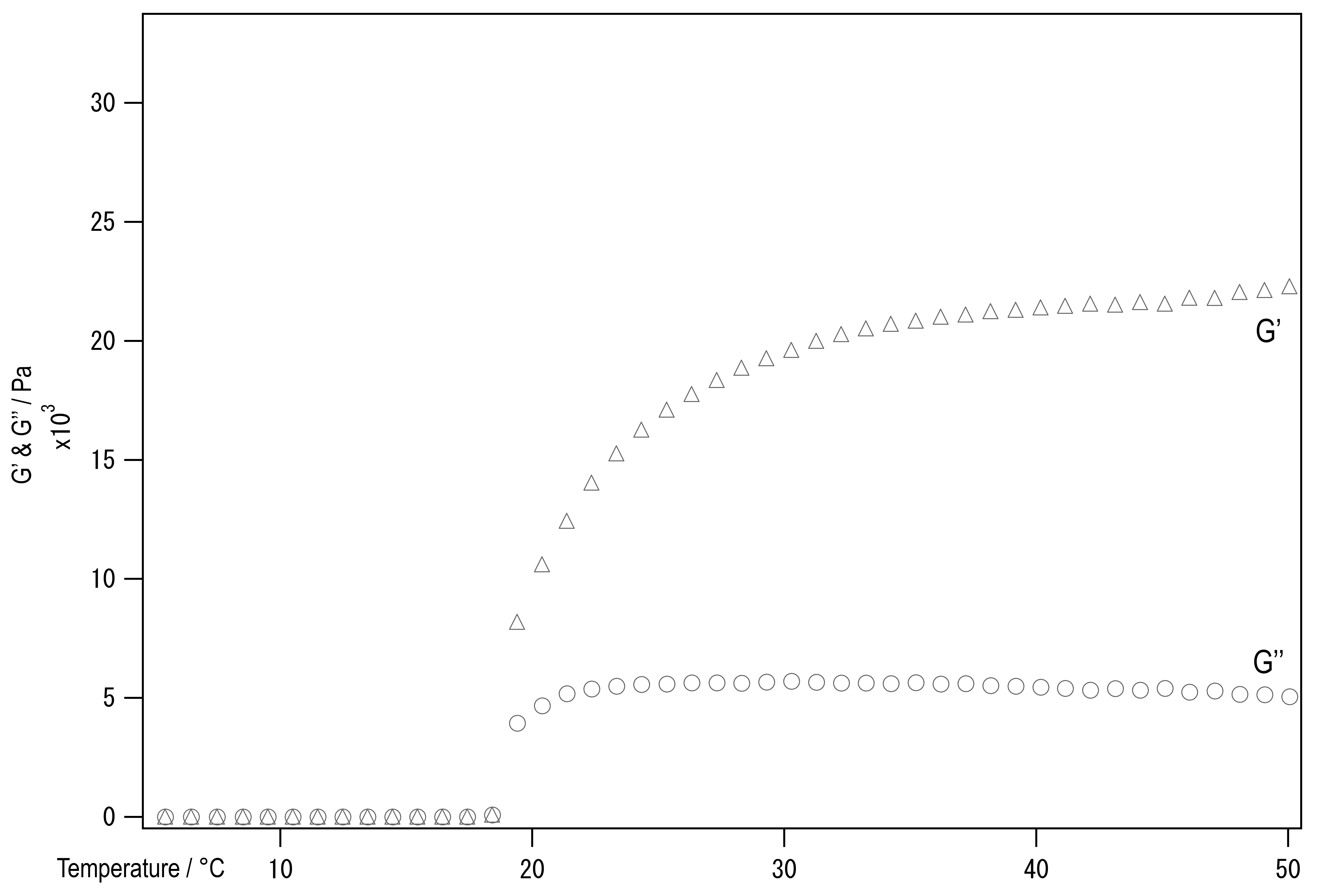

Supplement: Supplementary file 2 — Additional file 2. [file 40824_2022_337_MOESM2_ESM.png]
